# The construction of nursing performance evaluation model in community health service center based on the balanced scorecard and hygiene factors

**DOI:** 10.1038/s41598-022-26334-4

**Published:** 2022-12-16

**Authors:** Guiyun Yang

**Affiliations:** WuHan Fourth Hospital, Wuhan, Hubei China

**Keywords:** Health care economics, Health care, Health occupations

## Abstract

To develop and design a nursing performance evaluation model for community health service centers for nursing performance management. Preliminary evaluation indicators were constructed through literature retrieval and research. Applying the Delphi method, 20 experts were invited to conduct two rounds of questionnaire consultation and indicator importance scoring. The primary indicators use the Delphi method to determine the weights, and the secondary indicators use the AHP method to determine the weights. The nursing unit evaluation model (including 30 indicators), the nursing staff evaluation model (including 21 indicators), and the performance evaluation hygiene factors model (including 5 indicators) were constructed. The recovery rate of the expert questionnaire was 100%, and the authority coefficient was 0.768. The degree of coordination was in line with the standard, and the consistency of the judgment matrix of the analytic hierarchy process was acceptable. The model is scientific and innovative, which adapts to the work characteristics and development needs of community health service centers, and provides a practical tool for nursing performance evaluation.

## Introduction

### Research background and importance

Community health service is an important health cause in China, and China has invested a lot of money and human resources^1^. Community health service centers rely on national financial appropriations to undertake a large number of basic public health services in their jurisdictions. They are subsidized by the government, lack market competition, and do not face severe survival and development problems. It is very important to strengthen the management of human resources and improve the operation efficiency of medical staff by carrying out performance evaluations. Some community medical managers put forward that establishing sound performance management and evaluation mechanisms can comprehensively improve the Service quality and management effectiveness of community health service centers^2^. When performance management is not used in community health service centers, many mechanisms are unreasonable and lack rigor, which can easily discourage the work enthusiasm of medical staff and is not conducive to forming a good doctor-patient relationship^2^. Since 2019, the performance management policy of community health service centers has changed. Guangdong and Shandong provinces have allowed community health service centers to extract rewards and pay performance pay to employees^3^. The policy, which will be gradually promoted in all provinces and cities in China, puts forward higher requirements for performance appraisal.

Currently, Balanced scorecard (BSC) has been gradually promoted from enterprises to hospital nursing performance assessment in China^4^. The Balanced Scorecard is a performance evaluation tool established by Harvard Business School professor Robert Kaplan and others in the early 1990s, and Harvard Business Review lists it as the most influential strategic tool in the past 75 years^5^. New research has emerged in recent years^6,7^. The management tool is well-established in hospitals in China. Wang and Yijun et al.^8,9^ used the balanced scorecard to establish a nursing unit assessment system; Zhang et al. established an individual assessment system for nurses^10^; Yang and Ma et al.^11,12^ carried out the performance assessment of head nurses, improving the quality of nursing and patients' satisfaction. The hospital nursing performance assessment based on BSC reported in the above literature uses a three-level index system, with some assessment indicators reaching more than 50^8–10^, which is complicated to calculate. With full-time performance management personnel, BSC can be successfully applied by relying on the hospital’s developed information management system to collect performance appraisal data.

There are significant differences between hospitals and community health service centers in China. Community health service centers are primary medical institutions, mainly responsible for preventing and treating common diseases and providing public health services for community residents. They do not carry out complex surgical operations and medical treatment, and the number of nurses is small, usually between 30 and 40. The medical information system of the community health service centers are simple, and the full-time management personnel are few, so the performance index system of the hospital cannot be used directly. In recent years, there have been literature reports on the performance evaluation of community health service centers in China. Most use the index system jointly by doctors, nurses, and community health service centers management departments^13,14^. The index evaluation is mainly based on medical work, and the nursing performance management is very weak. Some studies have reported that in the performance evaluation index system used by community health service center, the evaluation indicators for individual nurses are only 6^15^. Because the work of doctors and nurses is completely different, doctors are mainly responsible for disease diagnosis and treatment. In contrast, nurses undertake more nursing services, including care services, health education and continuing care. The assessment indicators jointly used by doctors and nurses cannot comprehensively assess the work performance of nurses or nursing teams, and the indicators lack completeness and effectiveness.

Nursing staff accounts for more than half of the medical institution employees^16^, and they are the main practitioners in community health service centers and the main object of performance management. With the development of national hospital management, the establishment of a systematic, scientific, objective, and operable nursing performance evaluation and evaluation system is required for the reform of the medical system and the development of nursing^17^. The nursing performance management evaluation mechanism can stimulate the enthusiasm of nurses, improve nursing quality and patient satisfaction, can effectively promote the level of nursing management, and achieve good results^18^. Based on the application of BSC in hospitals in China, it is very important to construct an applicable nursing performance assessment model for community health service centers and carry out nursing performance assessment. However, according to the search of public literature reports, there are many nursing performance appraisal in hospitals in China, but there are few researches on nursing performance appraisal in community health service centers, and there are still research gaps.

Performance appraisal is easy to cause dissatisfaction of the subject. According to the research conducted by Li Wenjun on 220 medical staff^19^, only 20% of them expressed their understanding of performance evaluation, and 40% of them were not satisfied or dissatisfied with the way of performance evaluation. According to Herzberg's motivator-hygiene theory, if the hygiene factors cannot be satisfied, it will make employees dissatisfied and slow down their work. It cannot motivate people, but can maintain the enthusiasm of people and maintain the status quo of work, and is a factor related to the work environment^20^. Shen Xijuan et al. reported that hygiene factors were used to reduce the dissatisfaction of nurses with daily work^21^. If the performance assessment indicators and hygiene factors are designed and implemented simultaneously, it can not only promoted the scientificity of the indicators, but also reduce the dissatisfaction of the assessed personnel, which can effectively promote the development of nursing performance assessment. According to the literature search, few researchers pay attention to applying hygiene factor theory in performance appraisal.

### Topic of research paper

Based on the balanced scorecard and the theory of hygiene factors, the Delphi method was used to revise the assessment indicators, and the analytic hierarchy process was used to calculate the weight of the indicators. A new assessment model and a hygiene factor model were constructed for the nursing performance assessment of community health service centers. This study solves the problem that there is no applicable nursing performance appraisal model in community health service centers, applies the theory of hygiene factors to performance appraisal, and carries out a new scientific exploration.

### Research content and framework

This study used the four dimensions of BSC: customer, finance, internal operation and staff growth and development as the direction of performance assessment. Based on the community nursing contents such as basic nursing, health education, chronic disease nursing, rehabilitation nursing and resident health care, four first-level assessment indicators and several second-level assessment indicators were initially constructed. Several hygiene factor indexes were constructed according to the theory of hygiene factors. Experts were invited to use Delphi method to score, revise and screen the indicators, and the analytic hierarchy process was used to calculate the weight of the indicators and build the model. The Delphi method is a method of forming the results of a consultation. Founded by the RAND Corporation of the United States in 1946, it anonymously solicits opinions from experts through letter inquiries, organizes, summarizes, and gives feedback on opinions, and then conducts new letter inquiries. After repeated rounds of inquiries, opinions gradually tend to be consistent^1^. Because the Delphi method can fully use experts' experience and knowledge, the final conclusion is reliable and unified. This method has been widely used in prediction, decision making and the establishment of various evaluation index systems over the years^22^, and this method is also used in this study. Since community health service centers are primary medical institutions, the assessment indicators can not be complicated, which can reflect the main work of nursing and facilitate statistical accounting. Performance appraisal objects include individual nurses and nursing teams. This study constructed two performance appraisal models: nursing unit and individual nurses.

Analytic Hierarchy Process (AHP) is a hierarchical weighting method proposed by American operations researcher Thomas Sadie in the 1970s, which can transform semi-qualitative and semi-quantitative problems into quantitative calculations to guide decision-making^23^. AHP constructs a judgment matrix by comparing with each other and using a relative scale. In this way, the calculated indicator weights have higher accuracy. This can digitize and model the subjective judgment of experts, and improve the guidance and operability of the model. Index weight can reflect the importance of an index in the whole system. The index with high weight value can influence the assessment result and guide employees to complete it, reflecting the guidance direction of the assessment. Take one example^12^: Shaoxing Central Hospital carried out a nursing performance assessment, and the first-level indicators and weights were: cost-effectiveness (weight 0.2), nursing quality and safety (weight 0.4), learning and growth (weight 0.2), and customer satisfaction (weight 0.2). The weight value of nursing quality and safety was the highest, and nurses made more efforts to complete this index. After 2 years of performance assessment, nursing quality and safety improved compared to previous years (*P* < 0.05). If you do not set the weight, the formation of all assessment indicators equals the loss of the guiding role of some important indicators. The weight can be set by subjective assignment method based on expert consultation and objective assignment method based on data calculation^24^. 80% of the consulting experts in this study held nursing management positions in community health service centers, with rich management experience, and were familiar with the weights of the four first-level indicators. The subjective assignment method was used. Because of the large number of second-level indicators, it is difficult to concentrate and unify expert opinions, so AHP is used to calculate the weight.

Using the above theoretical tools and methods, we completed the construction of nursing performance appraisal model. The specific contents are as follows:

## Objects and methods

### Objects

Consultants are specialists in nursing and management. Nursing experts: in community health service centers chief nurse or above or engaged in nursing management work for more than 15 years in charge of nurses. Management experts: Associate professor or above in the School of Hospital Management and School of Public Health of China’s key universities, with certain research on management theories and methods. The experts have a certain enthusiasm for this research and are willing to answer the expert consultation questionnaire, a total of 20 people, all of whom are outside the hospital. Referring to other similar studies, considering the expert consultation cost provided by this research fund and the number of experts familiar with community nursing management in Wuhan, the number of experts is determined to be 20. The experts are recommended by the deputy director of the community nursing Committee of Wuhan Nursing Society. Researchers only know each expert's work unit, academic title, and job title, and other information, that will not be disclosed. Researchers and experts were consulted by E-mail correspondence, and the experts did not know each other's information. This study will not display any personally identifiable information and does not involve the privacy of consulting experts. The basic information of consulting experts is shown in Table 1.

**Table 1:** Basic information of consulting experts.

Project		Number of persons (person)	Percentage (%)
Gender	Female	19	95
	Male	1	5
The title status	Intermediate title	12	60
	Associate professor or deputy senior title	6	35
	Professor	2	10
Office	Community health service centers	16	80
	Hospitals	3	15
	Colleges and universities	1	5

### Set up a design team

The team consists of 8 nursing management and clinical nursing staff, including 3 nurse practitioners, 3 nurse-in-charge as well as 2 vice professor nurses. Among them, 4 carried out literature retrieval and data statistics, 2 carried out research on community health service centers, 1 was responsible for drafting assessment indicators and revising indicators according to expert opinions, and 1 carried out solicitation of experts. All the members worked in Wuhan Fourth Hospital, and they knew each other about their job positions and professional titles. The privacy of the 8 members was not involved in the research process.

### Establish preliminary evaluation indicators

In accordance with the spirit of these documents "Notice on Launching Community Service Improvement Project", "Community Health Service Quality Evaluation Index System (2015 Edition)" and "National Basic Public Health Service Project Performance Evaluation Guidance Plan" which are jointly issued by the National Health and Family Planning Commission and the State Administration of Traditional Chinese Medicine, the design team also combined the performance appraisal experience of the ChangQin Street community health service center managed by WuHan Fourth Hospital in recent years. After many investigations, discussions and revisions, two sets of evaluation indicators were initially designed for the nursing unit quarterly and the clinical nurse monthly.It includes four primary indicators, including satisfaction, work quality and quantity, scientific research and teaching, financial indicators, as well as several secondary indicators. In addition, hygiene factors measures for evaluation are designed in it. The design team completed the preliminary evaluation index design, and 20 experts were invited to use Delphi method to consult and revise the index. The design team of 8 people did not participate in the Delphi method of consultation.

### Design expert letter inquiry form and indicators assignment

The expert letter inquiry form is divided into three items, namely, the preliminary performance evaluation system, the expert's rating of the indicators recognition, and the expert's revision opinion. The expert recognition score is the Likert 5-level scoring method, which divides the importance of indicators in the evaluation into 5 levels: very important, important, general, usable, and unnecessary. The corresponding points are 5 points, 4 points, 3 points, 2 points, and 1 point. Experts' revision opinions are open-ended, and they can put forward revision opinions or supplement new indicators for each indicator.

### Consultation methods and indicators of screening

The preliminary designed evaluation system and hygiene factors measures will be distributed to all experts in the form of e-mails, and experts will score each indicator and may attach revision opinions. The indicators are filtered and revised based on the returned scores. Screening criteria: mean of importance assignment > 3.5, full score frequency > 0.2, and coefficient of variation < 0.2. Then the next round of consultation will be carried out. If the scoring results returned by the experts in the next round are calculated, all indicators meet the adoption standards, and the coordination coefficient is significant after the test, the expert consultation will be completed.

### Determine indicator weights

The primary indicator weights are constructed using the Delphi method. The secondary indicators use AHP. The principles and methods are as follows: (1) Establish a hierarchical structure model, including the target layer, the criterion layer and the program layer. (2) We construct a judgment matrix, use the mean of the importance score value of each indicator given by experts, compare the importance of each factor in pairs according to Saaty's "1–9 scaling method", and build a comparative judgment matrix^25^. According to the basic principle of the analytic hierarchy process, we calculate the product of elements in each line of the comparative judgment matrix, the eigenvector value, and the nth root of it. n is the order of the matrix. The value of the nth root is normalized, and the number is changed into a decimal between 0 and 1, which becomes the weight value of the index. (3)The consistency test is carried out, and the eigenvalues, eigenvectors, and consistency test indexes of each judgment matrix are calculated. The consistency ratio is represented by CR, CR = CI/RI, CI is the consistency index, CI = (λmax − n)/(n − 1), where λ_max_ is the maximum characteristic root of the matrix, RI is the average random consistency index. It is related to the order of the matrix. The larger the order is, the larger the RI is. The RI value can be obtained by querying the statistics table^26^. If CR < 0.1, the matrix passes the consistency test, indicating that the weight determined is valid and the consistency of the judgment matrix is acceptable.

### Statistical method

The software SPSS 17.0 was used to analyze the data, and the degree of expert coordination was analyzed using Kendall's W coefficient of non-parametric test of multiple correlated samples, and the test level was α = 0.05. The AHP was analyzed using Yaahp 12.6 software. Yaahp was used to calculate the CR value and index weight of each judgment matrix.

### Research duration

Starting from April 2022, an 8-member design team was established to establish preliminary assessment indicators through literature research. 20 consulting experts were recruited in April 2022, the first round of consultation was completed on April 30, 2022, and the second round was completed on May 10, 2022. On May 20, 2022, expert consultation, index screening, statistical calculation, model construction and other work were completed to form the results and complete the research work.

### Medical ethics statement of this study

The research method follows the ICH-GCP, Chinese GCP, the Declaration of Helsinki and relevant national laws, carried out the study in accordance with the protocol approved by the ethics committee of Wuhan Fourth Hospital to protect the health and rights of the subjects.The ethics Committee of Wuhan Fourth Hospital approved the experimental protocol of this study on April 21, 2022, and issued an ethical review and approval document.All subjects in this study were informed and consented to participate in the study.

## Results

### Expert enthusiasm

The questionnaire recovery rate reflects the enthusiasm of experts. In the first round, 20 questionnaires were distributed, 20 were effectively recovered, and the recovery rate was 100%. After an interval of 15 days, 20 questionnaires were distributed in the second round. The experts in the second round were the same as those in the first round. 20 questionnaires were effectively recovered, and the recovery rate was 100%.

### Expert authority

This study invited 20 experts, including 2 with senior professional titles and 6 with deputy senior titles. Using the Delphi method proposed by Zeng Guang to assign the authority degree^27^, the authoritative degree of experts in this study q = (expert academic level coefficient q1 + index judgment coefficient q2 + proficiency coefficient q3)/3. The authority degree assignment coefficient of The Delphi method proposed by Zeng Guang is: Q1 is assigned according to the professional title of experts (1.0 for doctoral supervisor, 0.9 for master supervisor or professor, 0.7 for other senior titles, 0.5 for associate senior titles and 0.3 for others); Q2 evaluated experts' theoretical level, practical experience, peer review, and expert intuition (experts' high, medium, and low theoretical level were assigned 0.3, 0.2, and 0.1, respectively; experts’ high, medium, and low practical experience were assigned 0.5, 0.4, and 0.3, respectively; Both peer review and expert intuition were assigned 0.1); Q3 Evaluation experts' familiarity with indicators (very familiar, familiar, general, not very familiar, not familiar, successively assigned 1.0, 0.8, 0.5, 0.2, 0.0). In the two rounds of expert consultation, q = 0.768, q1 = 0.425, q2 = 0.89, q3 = 0.99. Expert authority level q > 0.7, which means acceptable.

### Expert consultation coordination and consistency

The degree of coordination of expert consultation is expressed by calculating the coordination coefficient W value according to Kendall's W harmony coefficient, and the W value is between 0 and 1. If *p* < 0.05 for the significance test of the coordination coefficient, it means that the coordination coefficient is significant after the test, and the evaluation results of the experts on the indicators are consistent. The degree of coordination of expert consultation is shown in Table 2.

**Table 2:** Coordination and consistency.

	Nursing unit indicators		Clinical nurse indicators		Hygiene factors	
	W	P	W	P	W	P
First consultation	0.350	*p* < 0.01	0.370	*p* < 0.01	0.117	*p* < 0.05
Second consultation	0.116	*p* < 0.01	0.106	*p* < 0.01	0.131	*p* < 0.05

### Screen indicators

The preliminary evaluation system includes 34 nursing unit evaluation indicators, 24 clinical nurse evaluation indicators, and 6 nursing performance evaluation health factor measures. After the first round of consultation, 4 nursing unit evaluation indicators, 4 clinical nurse indicators, and 1 health care factor indicator did not meet the screening criteria. The scores of the second round of consultation indicators all met the screening requirements, and no indicators were increased or decreased. Some experts propose revisions to the indicator presentation.

### Form indicators and model building

After 2 rounds of expert consultation and revision, the primary indicator weights were determined by the Delphi method and the weights of the first-level indicators were 0.2, 0.5, 0.2 and 0.1. Using the analytic hierarchy process, the secondary indicator weights were calculated. The consistency test showed that the consistency ratio results were all 0.00, with a ratio < 0.10, and the results could be used. Finally, the nursing unit performance evaluation model (Table 3), the clinical nurse performance evaluation model (Table 4), and the performance evaluation hygiene factors measures model are formed (Table 5).

**Table 3:** Nursing unit performance evaluation model.

Number	Primary indicators	Secondary indicators	Secondary indicators weight
1	Patient satisfaction and medical ethics (Weight is 0.2)	No patient complained effectively	0.0487
2		Patients send flags and thank you letters, refuse to receive red envelopes, medical security	0.0491
3		No violation of the community health service center's employee handbook	0.0510
4		Investigation of patient satisfaction in the department	0.0513
5	Quality and quantity of nursing (weight is 0.5)	Health education implementation rate	0.0305
6		Follow-up rate after discharge	0.0306
7		Rehabilitation nursing (chronic disease management) implementation rate	0.0308
8		Implementation rate of nursing workflow	0.0308
9		I–IV nursing adverse events	0.0311
10		Implementation rate of writing nursing documents	0.0312
11		Implementation rate of basic nursing	0.0312
12		Undamaged rate of first aid items	0.0317
13		Accuracy rate of patients identification	0.0317
14		Accuracy rate of risk evaluation of patients fall	0.0311
15		Accuracy rate of risk evaluation of patients with stress injury	0.0311
16		Accuracy rate of risk evaluation of patients get lost	0.0310
17		Accuracy rate of risk evaluation of patients commit suicide and self-injury	0.0310
18		Accuracy rate of pipeline slippage risk evaluation in patients	0.0313
19		Accuracy rate of handover of patients	0.0317
20		Nursing practice rate of critically ill patients	0.0334
21	Scientific research, teaching and education training (Weighting is 0.2)	Teaching and training for interns and specialized nurses	0.0335
22		Completion of continuing education credits in nursing units	0.0320
23		Nursing unit "three basic" and specialized theory examination pass rate	0.0335
24		Research projects and published papers of nursing unit (annual bonus points, papers published from statistical sources or above, + 1 point/paper, research projects approved by Wuhan Municipal Health Commission or above, + 3 points/paper)	0.0328
25		The nursing unit undertakes the whole community health service center nursing round, difficult case discussion or business teaching situation	0.0340
26		New specialist nurses or further studies in nursing units	0.0341
27	Equipment, consumables and finance (Weight is 0.1)	Maintenance of valuable instruments and equipment	0.0256
28		No improper or illegal operation and use of valuable equipment	0.0244
29		No device is lent or lost	0.0257
30		Medical consumables and drug expiration date management	0.0244

**Table 4:** Clinical nurse performance evaluation model.

Number	Primary indicators	Secondary indicators	Secondary indicators weight
1	Patient satisfaction (Weight is 0.2)	Patient satisfaction Survey	0.1032
2		No patient complained effectively	0.0968
3	Quantity, quality and ability of work(Weight is 0.5)	Implementation rate of basic nursing	0.0565
4		Nursing practice rate of critically ill patients	0.0586
5		Rehabilitation nursing (chronic disease management) implementation rate	0.0541
6		Health education implementation rate	0.0536
7		Nursing quality control supervision and inspection	0.0552
8		I–IV nursing adverse events	0.0549
9		The rate of specialty sensitive index value reaching the standard	0.0550
10		Evaluation of nursing core system and operation	0.0565
11		Personnel attendance	0.0557
12	Personal learning and development (Weight is 0.2)	Presided over nursing ward rounds and professional training classes	0.0301
13		Teach new employees or interns	0.0286
14		Obtain the qualification of a specialist nurse or undertake a specialist nursing operation	0.0283
15		Participate in department nursing quality control or nursing quality improvement project	0.0287
16		Win prizes in the nursing competition	0.0277
17		The community health service center's year-end evaluation is excellent	0.0276
18		Municipal and above nursing staff commendation	0.0290
19	Financial dimension (weight is 0.1)	No medical items were damaged or lost on duty	0.0334
20		No serious waste and private use of medical materials and drugs	0.0322
21		violation of operation procedures to damage valuable equipment	0.0345

**Table 5:** Performance evaluation hygiene factors measures model.

Number	Description of measures	Weight
1	The head nurse is the person in charge of evaluation and takes evasive measures. The average reward of nurses in the department is 1.3–1.5 times for their performance, or nursing department organizes nurses, doctors and patients to conduct multi-dimensional evaluation	0.1885
2	Implement performance feedback. The head nurse conducts feedback to the individual every month, gives affirmation and encouragement to the outstanding individuals, and help the unskilled ones	0.1971
3	The head nurse provides timely guidance and assistance to new employees who have difficulties in completing their work	0.1955
4	Assess fair and just measures. Set up the department examination group, the members of the examination group supervise each other and check the data	0.2061
5	Improve the evaluation system, organize the study of evaluation standards, and promote nurses to be familiar with the evaluation content	0.2129

### Data analysis

According to Sun Ruimin’s report^28^, the Delphi method questionnaire recovery rate of more than 70% indicates the goodness of the survey. The recovery rate of the two rounds of this study was 100%, and the enthusiasm of the experts was high. In the calculation of the authority coefficient of experts, 16 experts were hired from community health service centers in this study, accounting for 80%, and the weight of practical experience is high. The expert authority coefficient is greater than 0.7, and the result is considered acceptable^29^. In this study, the expert authority coefficient is q = 0.768, and the expert authority is acceptable. The situation reflected by the coordination coefficient was significant after the first round of the coordination coefficient test (*P* < 0.01), but there were 9 indicators that did not meet the adoption criteria, and the second round of consultation was implemented after modification. After the second round of the consultation coordination coefficient tests, it was significant (*P* < 0.01), and the index scores all met the adoption criteria. No index was deleted, and the consultation was stopped to form a result. The W value of the nursing unit and individual nurses in the second round of consultation was smaller than that in the first round, indicating that the degree of coordination of the evaluation of a single index by experts gradually increased^30^. The W value of the second round of consultation on health factors was greater than that of the first round, indicating that the degree of consistency of experts' evaluation of the overall indicators was increased.

## Conclusions and suggestions

### The advantages and applicability of the results of this study

Compared with the performance evaluation methods reported in the literature, the results of this study are more suitable for community health service centers. Mainly for: (1) In this study, the balanced scorecard theory was used to construct a model, instead of using the three-level index system commonly used in hospitals reported in the literature^8^, but adapting to the small scale of community health service centers, using about 20 two-level indicators. Simplicity and operability. (2) In this model, the weight of nursing work quality and quantity dimension is 0.50, accounting for 50% of the overall weight, and the nursing quality evaluation is the focus. It further conforms to the "Medical Quality Management Measures" implemented by the National Health and Family Planning Commission of China in 2016, that the medical quality management situation is an important basis for the performance evaluation of medical staff. (3) The model is adapted to the elderly, inconvenient, recurring disease and chronic disease management of patients admitted to the community health service centers, and is included in the corresponding indicators. Through performance appraisal, community care is promoted to focus on chronic disease management and health education and is committed to the healthy China development strategy. (4) The hygiene factor measure model of performance appraisal was established and recognized by consulting experts. In the two rounds of expert letter consultation of Delphi method, an additional survey was added to the questionnaire as "whether hygiene factors measures are beneficial to the implementation of performance evaluation". All 20 experts in the two rounds of consultation chose the option of "favorable", indicating that this item was highly recognized by experts.

### Implications and suggestions of the research results

The assessment model contains few public health indicators. The main reasons are as follows. The community health service centers in Wuhan where this study was conducted have all established public health departments and employed medical staff to carry out their work, with less participation of nursing staff and few nursing performance indicators. In the model, there were many indicators of basic nursing, clinical nursing and chronic disease management. At the same time, there was a lack of assessment indicators of family nursing, which indicated that community nursing work focused on the nursing of common diseases and the full-cycle management of chronic diseases. Nurses carried out few family nursing works. It is suggested that the community nursing work in Wuhan should be further developed in an all-around way, and the work of family nursing and nurses' home visits should be expanded to serve the community residents better.

### Research limitations

The study has the following limitations. The consulting experts were selected in Wuhan, and the opinions formed were somewhat regional. Due to the differences in the work of community health service centers in different regions and the different performance appraisal objectives, the research results may not be extended to other regions or the whole country.

## Data Availability

The datasets generated during and/or analyzed during the current study are available from the corresponding author on reasonable request.
